# Supplementary material for: Selective Modulation of Hippocampal Theta Oscillations in Response to Morphine versus Natural Reward
Source: Brain Sci. 2023 Feb 14;13(2):322. doi: 10.3390/brainsci13020322 (PMC9953863; doi:10.3390/brainsci13020322)
Supplement: Supplementary file 1 [file brainsci-13-00322-s001.zip › brainsci-2164727-supplementary.pdf]

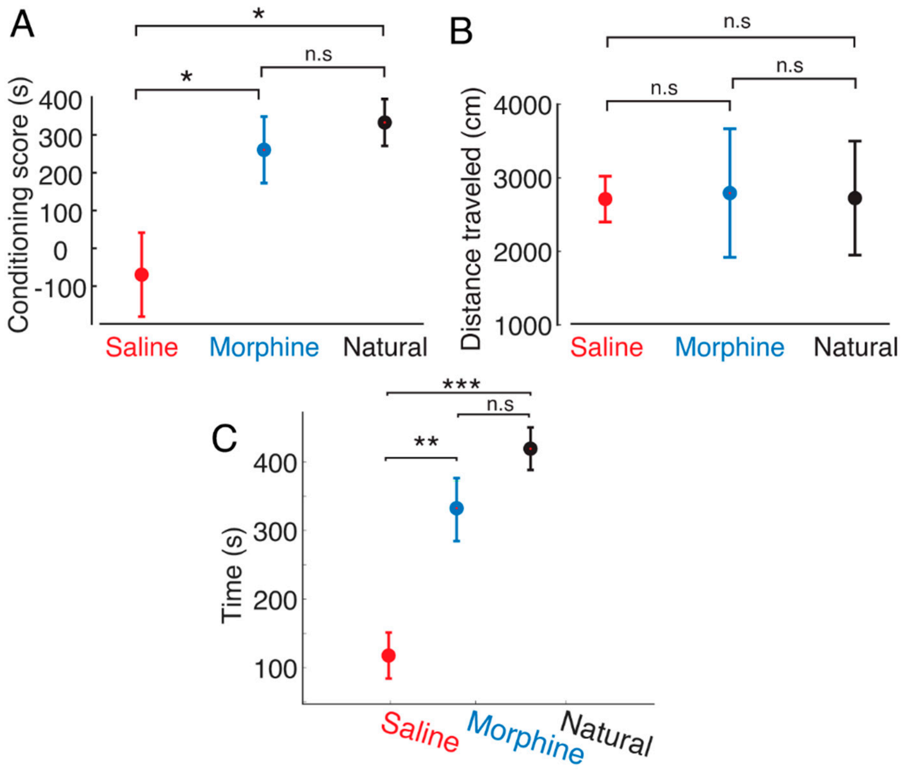

Supplementary Figure S1. (**A**) Comparing CPP score between saline, morphine, and Natural groups in the post-test phase; this figure shows that morphine- and food-induced CPP. In the natural group, animals received biscuits as a natural reward, and in the morphine group, the animal received morphine (5 mg/kg; s.c.) as a drug reward. (**B**) Comparing distance traveled among saline, morphine, and natural groups in the post-test phase; this figure indicates that there is no significant difference among these groups. **(C)** Comparing the time spent by animals in rewarded compartment during the post-test phase between saline, morphine, and natural groups.

Data represent mean ± SEM for 32 rats.

**P* < 0.05 as compared with the saline group

**P < 0.01 as compared with the saline group

***P < 0.01 as compared with the saline group
